# Can indicators of myocardial damage predict carbon monoxide poisoning outcomes?

**DOI:** 10.1186/s12873-021-00405-7

**Published:** 2021-01-15

**Authors:** Hitoshi Koga, Hideki Tashiro, Kouta Mukasa, Tomohiro Inoue, Aya Okamoto, Shougo Urabe, Shuuichirou Sagara, Kazumi Yano, Kouhei Onitsuka, Hisashi Yamashita

**Affiliations:** 1 Emergency Department, St. Mary’s Hospital, Kurume, Japan; 2 Division of Cardiology and Emergency Department, St. Mary’s Hospital, 422 Tsubuku-honmachi, Kurume, 830-8543 Japan

**Keywords:** Carbon monoxide poisoning, Carboxyhemoglobin, QT interval, QT dispersion, Troponin I

## Abstract

**Background:**

Carbon monoxide causes electrical, functional, and morphological changes in the heart. It is unclear, however, whether the indicators of myocardial damage can predict the patient’s prognosis after carbon monoxide poisoning. This retrospective study aimed to investigate the relationship between the carboxyhemoglobin level and electrocardiographic (ECG) changes and whether the ECG changes and troponin I levels are related to the patient’s prognosis after carbon monoxide poisoning.

**Methods:**

Carboxyhemoglobin, troponin I, and ECG parameters were measured in 70 patients with carbon monoxide poisoning. The QT and RR intervals were measured for each ECG lead in all patients, and the corrected QT interval and corrected QT dispersion were calculated.

**Results:**

The correlation between the maximum corrected QT interval and the carboxyhemoglobin level was significant (*P* = 0.0072, *R*^2^ = 0.1017), as were the relationships between QT dispersion and carboxyhemoglobin (*P* < 0.001, *R*^2^ = 0.2358) and the corrected QT dispersion and carboxyhemoglobin (*P* < 0.001, *R*^2^ = 0.2613). The multivariate logistic analysis showed that the significant predictors of sequential disability were corrected QT dispersion (*P* = 0.0042), and troponin I level (*P* = 0.0021).

**Conclusions:**

Patients’ prognosis following carbon monoxide poisoning can be predicted based on corrected QT dispersion and the troponin I level. Patients with myocardial damage should be monitored not only for their cardiovascular outcome but also for their neurological outcome and their prognosis.

## Background

Carbon monoxide is a colorless, odorless, non-irritating gas. It is produced endogenously in small amounts as a byproduct of the catabolism of heme molecules [1]. It can also be inhaled when hydrocarbon-containing fuels are not completely burned [1, 2]. Survivors of carbon monoxide poisoning may suffer neurological and psychiatric sequelae [1]. Carbon monoxide causes electrical, functional, and morphological changes in the heart [3]. The QT interval has long been known to vary significantly among the individual leads of a surface 12-lead electrocardiogram [3].

A potential clinical application of this inter-lead difference was proposed in 1990 by Day et al., who suggested that the inter-lead difference in the QT interval might provide a measure of repolarization inhomogeneity, which they called “QT dispersion” [3, 4]. Although it has been established that carbon monoxide induces electrocardiographic (ECG) changes and alterations of cardiac biomarkers [3, 5], it is unclear whether the indices of myocardial damage can predict the patient’s prognosis after carbon monoxide toxicity. Hence, this study aimed to investigate the relationship between carboxyhemoglobin and ECG changes and whether the ECG changes and troponin I levels are related to the prognosis of patients with carbon monoxide poisoning.

## Methods

The sample group of this retrospective study comprised patients suffering from carbon monoxide poisoning who had been admitted to St. Mary’s Hospital, Kurume, Japan for treatment between June 2013 and September 2019. St. Mary’s Hospital is the emergency medical center for southern Fukuoka Prefecture. Clear electrocardiograms were available for each patient and carbon monoxide poisoning had been confirmed by arterial blood analysis. Carboxyhemoglobin was measured in the ambulance and/or at admission, with the highest values being adopted. All patients whose blood and ECG could be analyzed using these techniques were included in the study. Encephalopathy was defined as the development or recurrence of symptoms such as difficulty concentrating, dementia, psychomotor retardation, Parkinson’s disease, and amnesia, which were either diagnosed by MRI at time of discharge or up to 6 weeks after admission. Sequential disability was defined as the major progressive outcomes resulting from carbon monoxide toxicity either at or after discharge. These include encephalopathy, cardiomyopathy, and death.

### QT interval measures and cardiac enzymes

All 12-lead electrocardiograms were obtained at a paper speed of 25 mm/s with standard lead positions. QT and RR intervals were measured on each electrocardiogram in all patients. The QT interval was measured from the beginning of the QRS complex to the end of the T wave. The QT intervals for each lead were measured and corrected for heart rate (QTc) using Bazett’s formula (QT/√RR) [6]. The QTc dispersion was the difference between the leads with the shortest and longest QTc intervals [4]. QT intervals were measured upon admission to the emergency department. Additionally, blood samples were obtained and the troponin I level was determined.

### Statistical analysis

Retrospective statistical analyses were performed using JMP and SAS university edition software (SAS Institute Inc., Cary, NC, USA). Results are presented as means ± SDs. An independent sample t-test was used to compare continuous variables, while Chi-squared tests or Fisher’s exact test were used for categorical variables. Spearman’s rank correlation coefficient was used to examine the relationships between carboxyhemoglobin levels and clinical variables.

Univariate and multivariate nominal logistic regression was performed to examine the relationships between patient prognosis and the level of carboxyhemoglobin, troponin I, and ECG parameters.

Additionally, ROC curve analysis was performed on these data, while the cut-off values were calculated using the Youden index. A value of *P* < 0.05 was considered to be statistically significant.

## Results

### Patients

Altogether, 70 patients (42 men, 28 women; mean age 52 ± 18 years) were included in the study, and their clinical characteristics are presented in Table 1. Two patients died after admission. Fifteen patients were diagnosed with encephalopathy either at or after discharge, whereas no patients exhibited cardiomyopathy at or after discharge.

**Table 1:** Patients’ characteristics

Male sex (%)	42 patients (60%)
Age (mean ± SD)	51.6 ± 18.2 years
Smoker	26 patients (37.1%)
Hypertension	10 patients (14.3%)
Diabetes mellitus	4 patients (5.7%)
Asymptomatic patients (%)	23 patients (33%)
Consciousness disorder on admission (%)	37 patients (53%)
Cardiopulmonary arrest (%)	3 patients (4%)
Hyperbaric oxygen therapy (%)	49 patients (70%)
Sequential disability (%)	15 patients (21%)
Deliberately carbon monoxide exposed patients (%)	20 patients (29%)
Carboxyhemoglobin level (%) (normal range 0.5–1.5)	21.8 ± 14.8
Troponin I (ng/ml) (normal range 0.00–0.09)	0.85 ± 3.36
Mean emergency department arrival time (min)	165 ± 148

### Correlation between carboxyhemoglobin and QT intervals

The correlation between maximum QT intervals and the carboxyhemoglobin levels was not significant.

**Fig. 1:**
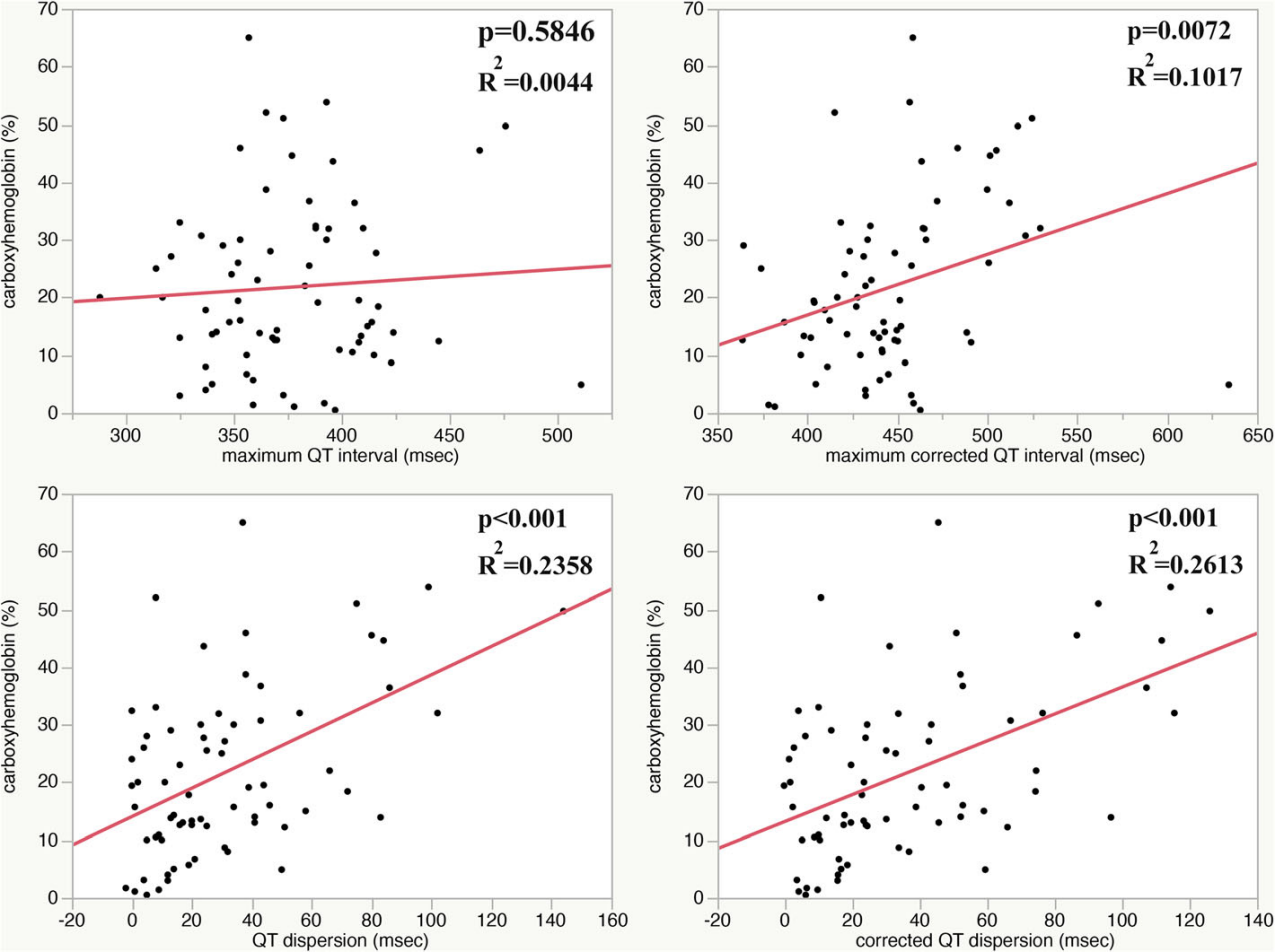
Relationship between the ECG parameters and carboxyhemoglobin. *Top left*. Relationship between the maximum QT interval and the carboxyhemoglobin level. *Top right*. Relationship between the maximum corrected QT interval and the carboxyhemoglobin level. *Bottom left*. Relationship between QT dispersion and the carboxyhemoglobin level. *Bottom right*. Relationship between corrected QT dispersion and the carboxyhemoglobin level. The relationships between the maximum QT interval and carboxyhemoglobin are not significant, whereas the other relationships are all significant

Conversely, the correlation between the maximum QTc interval and the carboxyhemoglobin level was significant (*P* = 0.0072, *R*^2^ = 0.1017; Fig. 1), as were the relationships between QT dispersion and carboxyhemoglobin (*P* < 0.001, *R*^2^ = 0.2358; Fig. 1) and the QTc dispersion and carboxyhemoglobin (*P* < 0.001, *R*^2^ = 0.2613; Fig. 1).

### Comparison between patients with and without a sequential disability

**Fig. 2:**
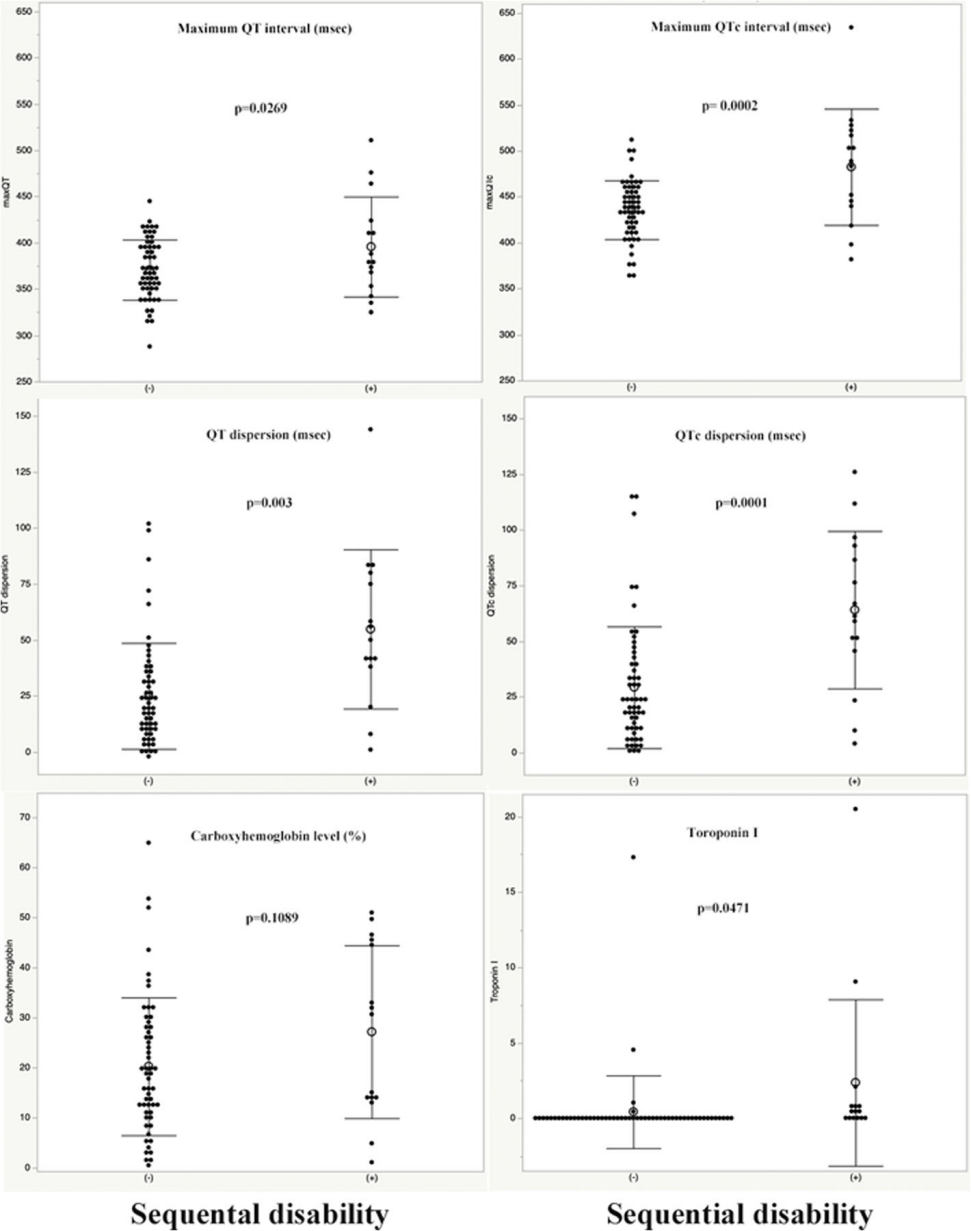
Top left: Comparison of the maximum QT interval in patients with a sequential disability (+) and without a sequential disability (−).Top right: Comparison of the corrected maximum QT interval in patients both with (+) and without a sequential disability (−). Middle left: Comparison of the maximum QT dispersion in patients both with (+) and without a sequential disability (−). Middle right: Comparison of the corrected QT dispersion in patients both with (+) and without a sequential disability (−). Bottom left: Comparison of the carboxyhemoglobin levels in patients both with (+) and without a sequential disability (−). Bottom right: Comparison of the troponin I level in patients both with (+) and without a sequential disability (−). There was no significant difference in the carboxyhemoglobin levels between the patients both with and without a sequential disability, whereas all the other comparisons were significantly different

Death, carbon monoxide encephalopathy, and cardiomyopathy at or after discharge were all considered to be sequential disabilities, although none of the patients in this study had cardiomyopathy. There were no differences in sex or age between patients with and without a sequential disability, while the number of patients taking tranquilizers of varying strengths or antidepressants and other drugs did not change either (Table 2). However, the maximum QT interval (*p* = 0.0269), the corrected maximum QT interval (*p* = 0.0002), the QT dispersion (*p* = 0.0003), and the corrected QT dispersion (*p* = 0.0001) were significantly longer in patients who had a sequential disability (Table 2, Fig. 2). Troponin I levels in patients with a sequential disability were greater than in those who did not (Table 2, Fig. 2). There were also no differences in carboxyhemoglobin levels between patients with and without a sequential disability (Table 2, Fig. 2).

**Table 2:** The comparison between the patients with and without sequential disability

	Sequential disability (+) *n* = 15	Sequential Disability (−) *n* = 55	p
Sex (Male: %)	67% (*n* = 10)	58% (*n* = 32)	0.5521
Smoker	20% (*n* = 3)	42% (*n* = 23)	0.1211
Hypertension	0% (*n* = 0)	18% (*n* = 0)	0.0745
Diabetes mellitus	0% (*n* = 0)	7% (*n* = 4)	0.2821
Consciousness disorder on admission	80% (*n* = 12)	38%(*n* = 21)	0.0040
Cardiopulmonary arrest	20% (*n* = 3)	0% (*n* = 0)	0.0083
Deliberately carbon monoxide exposed patients	47% (*n* = 7)	24% (*n* = 13)	0.0801
Hyperbaric oxygen therapy	60% (*n* = 9)	72% (*n* = 40)	0.3404
Hypokalemia	20% (*n* = 3)	16% (*n* = 9)	0.7405
Minor tranquilizer	13% (*n* = 2)	13% (*n* = 7)	0.9504
Major tranquilizer	7% (*n* = 1)	5% (*n* = 3)	0.8577
Antidepressant	7% (*n* = 4)	7% (*n* = 1)	0.9356
Other drugs	27% (*n* = 4)	29% (*n* = 16)	0.8538
Age (years)	53.9 ± 17.3	51.0 ± 18.5	0.5895
Mean emergency department arrival time	199.5 ± 192.0 min	156.0 ± 134.1 min	0.3159
Maximum QT interval (msec)	395.7 ± 54.1	370.6 ± 32.6	0.0269
Maximum QTc interval (msec)	482.6 ± 63.4	435.7 ± 32.1	0.0002
QT dispersion (msec)	54.8 ± 35.6	25.0 ± 23.6	0.0003
QTc dispersion (msec)	64.1 ± 35.4	29.3 ± 27.3	0.0001
Carboxy hemoglobin level (%)	27.1 ± 17.3	20.3 ± 13.7	0.1089
Troponin I (ng/ml)	2.4 ± 5.5	0.4 ± 2.4	0.0471

*Abbreviation*: *QTc* Corrected QT

### Nominal logistic analyses

The reference values for the maximum QTc interval, QTc dispersion, carboxyhemoglobin level, and troponin I level were established based on the Classification and Regression Tree.

The univariate logistic analysis showed that the significant predictors of sequential disability were consciousness disorder on admission (*P* = 0.0078), QTc dispersion (> 46 ms; *P* = 0.0003), high levels of carboxyhemoglobin (> 44%; *P* = 0.0044), and a high Troponin I level (> 0.36 ng/ml; *P* = 0.0003; Table 3). Multivariate logistic analysis was performed on QTc dispersion, carboxyhemoglobin levels, and troponin I levels. This indicated that the significant predictors of sequential disability were QTc dispersion (*P* = 0.0042) and troponin I level (*P* = 0.0021; Table 3).

**Table 3:** Findings of the univariate and multivariate analyses

	Univariate analysis		Multivariate analysis	
	Odds ratio (95% CI)	p	Odds ratio (95% CI)	p
Sex (M)	1.4375 (0.433–4.772)	0.5533		
Age (> 30)	1.714 (0.190–15.452)	0.6309		
Smoker	0.3478 (0.088–1.374)	0.1319		
Consciousness disorder	6.476 ((1.634–25.669)	0.0078		
Hyperbaric oxygen therapy	0.563 (0.171–1.851)	0.3493		
QTc dispersion (> 46 msec)	12.375 (3.260–46.970)	0.0002	12.333 (2.207–68.926)	0.0042
Carboxyhemoglobin level (> 44%)	8.666 (1.779–42.215)	0.0044	3.863 (0.433–34.467)	0.2262
Troponin I (> 0.36 ng/ml)	14.571 (3.463–61.311)	0.0003	19.709 (2.947–131.770)	0.0021

*Abbreviation*: *QTc* Corrected QT interval

**Fig. 3:**
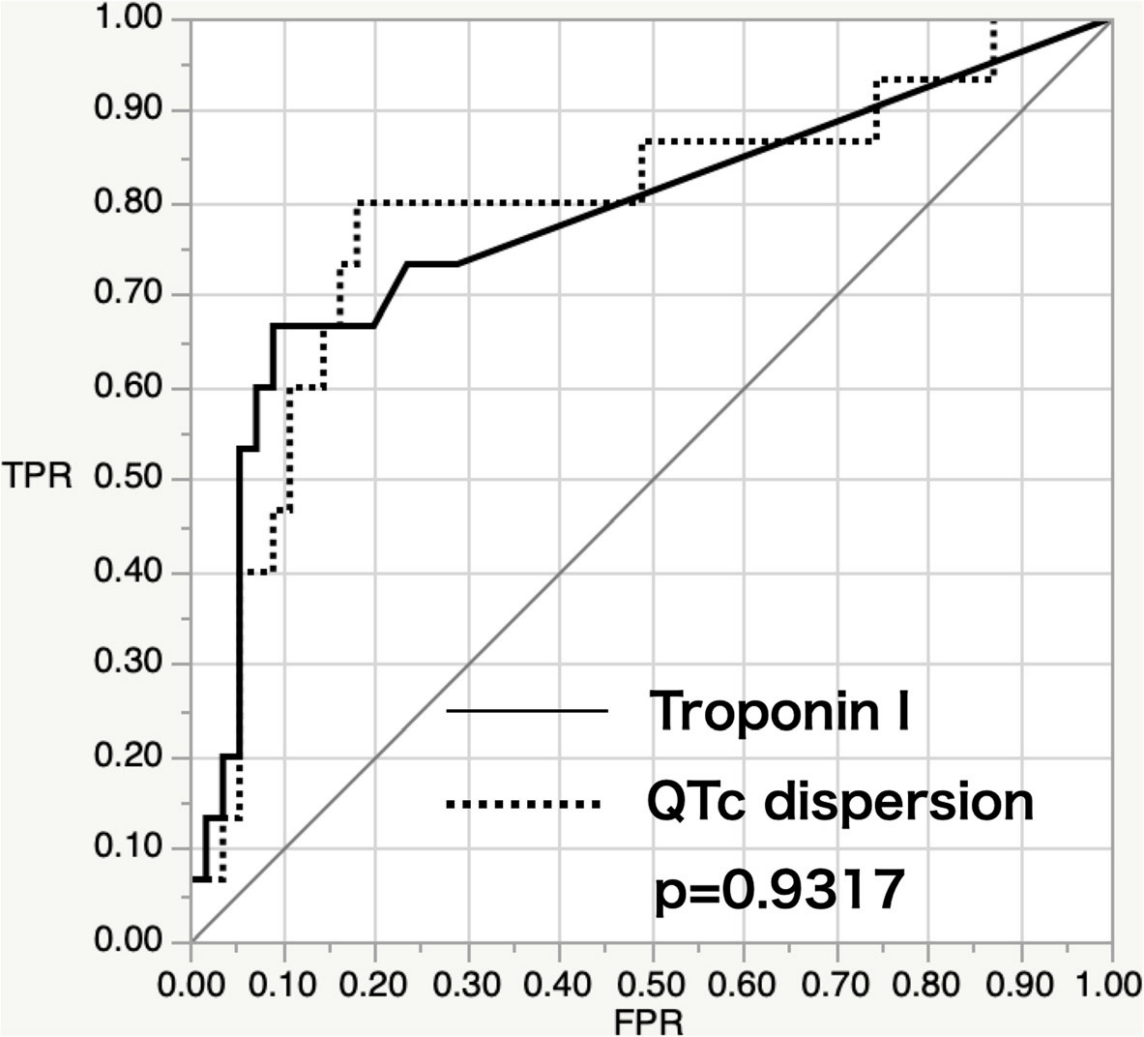
ROC curve analysis of QTc dispersion and troponin I level as significant predictors of a sequential disability. The curves show no significant differences between the two

ROC curve analysis was performed on QTc dispersion and troponin I levels (Fig. 3). The area under the curve was 0.789 for QTc dispersion and 0.780 for the troponin I levels. There was no significant difference between the two areas. The cut-off values were 45.6 ms for QTc dispersion and 0.12 ng/ml for the troponin I level.

## Discussion

Carbon monoxide poisoning, a serious health problem, is associated with a high incidence of severe morbidity and mortality. It causes myocardial toxicity and life-threatening arrhythmias. Carbon monoxide reduces the oxygen-carrying capacity of blood and binds with cardiac myoglobin, causing a rapid decrease in myocardial oxygen reserves [6]. Several studies have shown that carbon monoxide intoxication causes increased QT intervals and QT dispersions [6–8].

This study showed that QTc dispersion and carboxyhemoglobin are significantly related. Furthermore, both QTc dispersion and troponin I are predictors of sequential disability.

### Correlation between carboxyhemoglobin and QT intervals

The *QT interval* is an indicator of ventricular repolarization on the electrocardiogram. A *prolonged QT interval* reflects impaired myocardial refractoriness. *QT dispersion* reflects the physiological variability of regional ventricular repolarization. *Increased QT* dispersion is related to the heterogeneity of regional ventricular repolarization and is accepted as a marker for arrhythmia and sudden death [9]. Tanba et al. reported that the normal value of QTc dispersion in Japanese patients is 31 ± 11 ms [10].

In this study, the QTc interval and QTc dispersion correlated with the carboxyhemoglobin level. Hanci et al. also reported that the QTc interval and QTc dispersion show good correlations with carboxyhemoglobin [11]. Increased QTc dispersion and interval in carbon monoxide toxicity might be caused by carbon monoxide on the myocardium, which causes homogeneous impulse formation in the ventricles.

### Relationship between the prognosis of patients with carbon monoxide toxicity and cardiac markers

The univariate logistic analysis indicated that the predictors of sequential disability in patients with carbon monoxide toxicity were smoking, consciousness disorder, maximum QTc interval, QTc dispersion, carboxyhemoglobin, and troponin I. The multivariate analysis that was performed on QTc dispersion and on the levels of carboxyhemoglobin and troponin I revealed that QTc dispersion and troponin I were both significant predictors of sequential disability. Note that the multivariate analysis did not find that carboxyhemoglobin was related to a poor outcome. Furthermore, the areas under the curve were not significantly different.

However, these indicators might be able to predict the prognosis of patients with carbon monoxide toxicity. Conversely, although the prognosis of patients with carbon monoxide toxicity depends on the length of exposure and its concentration, carboxyhemoglobin was not necessarily related to exposure time or the concentration of the carbon monoxide inhaled.

Because patients with carbon monoxide toxicity usually receive treatment immediately after exposure, carboxyhemoglobin was not measured at its peak concentration.

Hampson et al. found that the level of carboxyhemoglobin in the blood was a poor predictor of the clinical prognosis of patients with carbon monoxide poisoning [12].

Moreover, mortality was associated with the absolute difference in carboxyhemoglobin [12]. Satran et al., however, reported that moderate-to-severe carbon monoxide poisoning causes myocardial injury when assessed by electrocardiography or biomarkers [5].

Because carbon monoxide binds to hemoglobin with high affinity, exposure to carbon monoxide, even in low concentrations, profound tissue hypoxia [13, 14]. Thus, carbon monoxide might cause neurological injury and likely contribute to myocardial injury as well. Although there were no patients with cardiomyopathy at/after discharge in this study, the indices of myocardial injury could lead to neurological injury or even mortality. The mechanisms described influence this result. In this study, hyperbaric therapy was not found to be significant by the univariate logistic analysis. However, patients with severe symptoms as a result of serious carbon monoxide poisoning often receive hyperbaric therapy. Therefore, this result may be biased.

### Limitations

The primary limitations of this study were the small sample population, single-center design, and retrospective nature. Additionally, because our subjects included patients who had deliberately as well as accidentally exposed themselves to carbon monoxide, several patients’ exact exposure time could not be precisely calculated. Despite these limitations, the study may act as a good basis for further study of the topic. The findings must be confirmed in prospective, multicenter studies with larger populations.

## Conclusion

The prognosis for patients with carbon monoxide poisoning may be predicted based on the QTc dispersion value and the troponin I level. Patients with myocardial damage should be monitored not only for their cardiovascular outcome but also for their neurological outcome and their prognosis.

## Data Availability

The datasets used in the current study are available from the corresponding author on reasonable request.

## Abbreviations

ECG: Electrocardiogram
QTc: Corrected QT interval
ROC: Receiver operating characteristic
